# Philosophical foundations for digital ethics and AI Ethics: a dignitarian approach

**DOI:** 10.1007/s43681-021-00040-9

**Published:** 2021-02-26

**Authors:** Robert Hanna, Emre Kazim

**Affiliations:** 1 Boulder, USA; 2 Computer Science, University College London, London, UK

**Keywords:** Human dignity, Ethics, Morality, Artificial intelligence, Machine learning, Digital, Data, Personhood, Agency

## Abstract

*AI Ethics* is a burgeoning and relatively new field that has emerged in response to growing concerns about the impact of artificial intelligence (AI) on human individuals and their social institutions. In turn, AI ethics is a part of the broader field of digital ethics, which addresses similar concerns generated by the development and deployment of new digital technologies. Here, we tackle the important worry that digital ethics in general, and AI ethics in particular, lack adequate philosophical foundations. In direct response to that worry, we formulate and rationally justify some basic concepts and principles for digital ethics/AI ethics, all drawn from a broadly Kantian theory of human dignity. Our argument, which is designed to be relatively compact and easily accessible, is presented in ten distinct steps: (1) what “digital ethics” and “AI ethics” mean, (2) refuting the dignity-skeptic, (3) the metaphysics of human dignity, (4) human happiness or flourishing, true human needs, and human dignity, (5) our moral obligations with respect to all human real persons, (6) what a natural automaton or natural machine is, (7) why human real persons are not natural automata/natural machines: because consciousness is a form of life, (8) our moral obligations with respect to the design and use of artificial automata or artificial machines, aka computers, and digital technology more generally, (9) what privacy is, why invasions of digital privacy are morally impermissible, whereas consensual entrances into digital privacy are either morally permissible or even obligatory, and finally (10) dignitarian morality versus legality, and digital ethics/AI ethics. We conclude by asserting our strongly-held belief that a well-founded and generally-accepted dignitarian digital ethics/AI ethics is of global existential importance for humanity.

In the realm of ends everything has either a *price* or a *dignity* (*Würde*). What has a price can be replaced by something else as its *equivalent*; what on the other hand is raised above all price and therefore admits of no equivalent has dignity. What is related to general human inclinations and needs has a *market price*; that which, even without presupposing a need, conforms with a certain taste, that is, with a delight in the mere purposeless play of our mental powers, has an *affective price* (*Affectionpreis*); but that which constitutes the condition under which alone something can be an end in itself has not merely a relative worth, that is, a price, but an inner worth, that is, *dignity*. Now, morality is the condition under which alone a rational being can be an end in itself, since only through this is it possible to be a lawgiving member in the realm of ends. Hence morality, and humanity insofar as it is capable of morality, is that which alone has dignity [1], p. 84.

## Introduction

AI ethics is a burgeoning and relatively new field that has emerged in response to growing concerns about the impact of artificial intelligence (AI) on human individuals and their social institutions [2–4]. In turn, AI ethics is a part of the broader field of digital ethics, which addresses similar concerns generated by the development and deployment of new digital technologies, including of course AI, big data analytics, and blockchain technologies [5]. Here, we tackle the profound worry that digital ethics in general, and AI ethics in particular, lack adequate philosophical foundations. The field is currently a big tent—covering questions of legality and compliance [6], speculative futurism with dystopian or utopian implications, and even moral panic—without an overarching set of basic concepts and principles [7]. In what follows, we formulate and rationally justify some basic concepts and principles for digital ethics and AI ethics. These concepts and principles, in turn, are all drawn from a broadly Kantian theory of human dignity [8].

But, backpedalling for a moment,  does *human dignity* really exist? If so, then what is its nature and how is that nature grounded, what are its essential moral implications, how do we know them, and how can this dignitarian knowledge be applied in real-world political contexts? No questions could be more important for humanity. Therefore, it is a rational human imperative to provide a clear, distinct, consistent, complete, and true—or at least philosophically intelligible, defensible and plausible—theory of human dignity. Obviously, we cannot undertake a full-dress presentation and defense of such a theory here. But here are capsulized versions of the answers we would give to those questions.

First, human dignity really exists because (i) no one, not even a dignity-skeptic, could give their actual or possible rational consent to being treated either as a mere means to someone’s ends (i.e., their desires or goals) or as a mere thing, and (ii) its being absolutely impermissible to treat any people (including oneself) either as a mere means or as a mere thing is an essential property of human dignity.

Second, human dignity is the absolute, non-denumerably infinite, intrinsic, and objective value of human real persons as ends-in-themselves, and human real personhood is metaphysically grounded in an essentially embodied, unified set of innate cognitive, emotional, and practical capacities present in all and only those human animals possessing the essentially embodied neurobiological basis of those capacities.

Third, the essential moral implications of human dignity are an hierarchically-ordered set of (either absolutely or *ceteris paribus*) universal moral principles specifying ways of always treating all human real persons with sufficient respect for their human dignity, the essence of which is the absolutely universal obligation never to treat any human real person (including oneself) as a mere means or as a mere thing, in a thoroughly nonideal natural and social world.

Fourth, human dignity and its essential moral implications are known by a multifaceted systematic method that includes (i) essentially reliable a priori moral intuitions of basic principles supplemented by logical rationality and reasoning, (ii) fairly reliable cognitive and practical constructive knowledge of non-basic principles under those basic principles, (iii) considered moral judgments in real-world contexts and in thought-experiments by way of applying and further specifying those basic and non-basic principles, and (iv) empathetic intersubjective moral phenomenology.

Fifth and finally, this dignitarian knowledge can be applied in real-world sociopolitical contexts only by enacting human dignity: that is, only by means of designing, creating, and sustaining all and only specifically dignitarian social institutions that Michelle Maiese and Robert Hanna have called *constructive, enabling* social institutions [9], chs. 1–3, and 6–7.

It should be already obvious that the theory of human dignity we have just capsulized is *Kantian* in philosophical inspiration. But although we are philosophically inspired by Kant’s writings, this theory of human dignity is *neither* intended to be an interpretation of Kant’s writings, *nor* in any way restricted by the requirement to remain consistent with or defend any of Kant’s own doctrines (for example, his alleged noumenal realism, hatred of emotions, moral formalism and rigorism, political liberalism, etc.) or his personal prejudices (for example, his alleged racism, sexism, xenophobia, etc.). Thus the theory of human dignity we are presenting is Kantian, but not so *damned* Kantian. This is a spin on Josiah Royce’s pithy definition of idealism: ‘‘the world and the heavens, and the stars are all *real*, but not so *damned* real” [10], p. 217]. In other words, the theory of human dignity that we are presenting involves a creative use of some Kantian ideas that are also independently defensible, and it diverges from either Kant’s own writings or orthodox Kantianism *whenever* that is required by attentiveness to manifest reality and/or critical reflection. In view of the social-institutional facts that one of us has called *the Kant wars*, one element of which is a widespread *anti-Kantian bias* in contemporary philosophy [11], it is (unfortunately) necessary to make this point explicitly. And to emphasize that point, we will call it *the broadly Kantian theory of human dignity*.

In view of the broadly Kantian theory of human dignity, we are postulating the existence of human real persons who inherently possess human dignity, and also the reality of their free agency—including free will, autonomy, and responsibility—as basic premises of digital ethics and AI ethics. Human real persons who inherently possess human dignity, and human real persons alone, design, develop, produce, adopt, and deploy digital technology and computers, by means of their creative agency and their freely willed choices. Therefore, human real persons who inherently possess human dignity, and human real persons alone, are deeply morally or non-morally responsible for whatever fate the digital future may bring.

We fully recognize that moral philosophy, social philosophy, political philosophy, and the metaphysics of human nature that grounds all these subjects, are all highly contentious and controversial; and also that software designers and/or computer engineers may want to avoid the seemingly esoteric and irrelevant abstractions of philosophers and go straight to designing and implementing algorithms that meet legal requirements. But since a leading motivation behind our project is to avoid *technological determinism*—i.e., the view that technology develops autonomously according to an internal logic and forces a prescribed social change—which sharply contrasts with our broadly Kantian dignitarian conception of an ordered and humane digital reality based on morally guided, rational, and autonomous choices—then we should not let technological-determinist tendencies frighten us into *avoiding* the task of explicit formulating and rationally justifying basic concepts and principles of digital ethics/AI ethics.

Because we are especially interested in articulating a cogent and coherent research program that (at least in principle) could unify the entire field of digital ethics/AI ethics, our argument is designed to be relatively compact and easily accessible, and is presented in ten distinct steps: (1) what “digital ethics” and “AI ethics” mean, (2) refuting the dignity-skeptic, (3) the metaphysics of human dignity, (4) human happiness or flourishing, true human needs, and human dignity, (5) our moral obligations with respect to all human real persons, (6) what a natural automaton or natural machine is, (7) why human real persons are not natural automata/natural machines: because consciousness is a form of life, (8) our moral obligations with respect to the design and use of artificial automata or artificial machines, aka computers, and digital technology more generally, (9) what privacy is, why invasions of digital privacy are morally impermissible, whereas consensual entrances into digital privacy are either morally permissible or even obligatory, and finally (10) dignitarian morality versus legality, and digital ethics/AI ethics. We conclude by asserting our strongly-held belief that a well-founded and generally-accepted broadly Kantian dignitarian digital ethics/AI ethics is of global existential importance for humanity.

## What “digital ethics” and “ai ethics” mean

Anything *X* counts as “digital technology,” or as “artificial intelligence” (aka AI), if and only if *X* is a machine, or a proper part of a machine, that processes information according to the logical rules of Turing-computation [12,13], aimed at some end or purpose. An “algorithm” is any well-defined, finite information-processing sequence according to the rules of Turing-computation. Algorithms are implemented by artificial automata or artificial machines—aka “computers”—and by digital technology more generally, of many different kinds, built by us out of many different kinds of materials. The end or purpose of any algorithm—say, to solve a class of problems, or to perform a calculation, or to yield some other sort of result (aka “optimization”)—is pre-set by human persons, i.e., by us. Therefore, all artificial automata or artificial machines—all computers—and all digital technology more generally, are nothing more and nothing less than tools for information processing according to the logical rules of Turing-computation, created by human persons for specifically human ends and purposes.

Ethics is the domain of our basic individual and social commitments, and our leading ideals and values. Morality, in turn, is the attempt to guide human conduct by rationally formulating and following principles or rules that reflect our basic personal and social commitments and our leading ideals and values; and morality is the *core* of ethics. Therefore, digital ethics is the attempt to guide human conduct in the design and use of digital technology in general, and AI ethics is the attempt to guide human conduct in the design and use of artificial automata or artificial machines, aka computers, in particular, by rationally formulating and following principles or rules that reflect our basic individual and social commitments and our leading ideals and values.

## Refuting the dignity-skeptic

The dignity-skeptic is anyone who, for any reason whatsoever, denies the real existence of human dignity. But if one asked the dignity-skeptic the following question,

“Would you or could you rationally consent to being summarily beheaded *merely* because of your skin pigmentation, ethnicity, gender, sexual orientation, ability or disability, religious beliefs, or political beliefs?, yes or no, please give reasons for your answer, and please reply in all sincerity,”

then if the dignity-skeptic complied with those requests, they would obviously answer “of course *not!*” But any reason sincerely provided by the dignity-skeptic that is sufficient to explain *why not*, would be necessarily equivalent to appealing to the skeptic’s *own* human dignity, since it would necessarily involve the absolute impermissibility of *themselves* being treated by someone as mere means for any purportedly sufficient reason that singles them out only as a mere token of some identity-type, thus reducing *themselves* to being a mere thing under that type. And its being absolutely impermissible to treat any people (including oneself) either as a mere means or as a mere thing is an essential property of human dignity. Therefore, the dignity-skeptic’s answer would entail or presuppose the real existence of human dignity in at least *themselves*. So the dignity-skeptic’s answer is self-refuting, and human dignity really exists.

## The metaphysics of human dignity

Given that human dignity really exists, then *what is it*? To be sure, there are a great many existing legal documents and sets of principles for digital ethics/AI ethics, that *appeal* to the notion of human dignity, but, as far as we know, there are none (so far) that adequately *explain* it [14, 15]. In the absence of serious metaphysics, however, the appeal to human dignity is nothing but hand-waving in the direction of such an explanation. Hence, although software designers, computer engineers, and policy-makers may not feel the need to engage in the formulation and rational justification of the first principles of such a moral metaphysics, or to explore the various implications of a dignitarian moral and political framework, it is nonetheless absolutely necessary for *someone* to undertake this task [7, 16, 17]. Because this essay is designed to be relatively compact and easily accessible, we will make no attempt to do a critical survey of the recent and contemporary philosophical literature on human dignity. But the footnotes for Adam Etinson’s recent article, “What’s So Special About Human Dignity?” [18], are useful in this regard, and for historical analyses of the concept of dignity, Remy Debes’s recent edited collection, *Dignity: A History*, [19] and his blog-post-style article “Dignity is Delicate” [20], are similarly useful.

According to our broadly Kantian theory of human dignity, human dignity is the absolute, non-denumerably infinite, intrinsic, and objective value of *human real persons* as ends-in-themselves, and human real personhood is grounded in a unified set of innate cognitive, emotional, and practical capacities present in all and only human animals possessing the essentially embodied neurobiological basis of those capacities. Some human animals are born permanently lacking this essentially embodied neurobiological basis or have suffered its permanently destruction by accident, disease, or violent mishap, and therefore *some* human animals do not have human dignity because they are not human real persons. So not necessarily all human animals are real persons. Conversely, not necessarily all real persons are human: it is really possible for there to be real persons belonging to other animal species, whether on the Earth or other planets. If so, then they will have dignity too.

Nevertheless and in any case, *you are a real person, and so are we*. And so is every other living organism that is capable of fully understanding those words, feeling their normative force, and then choosing and acting under the guidance of that normative force. Neither logically possible or conceivable non-animal persons, disembodied persons, or divine persons, nor actual artificial persons (personae) or actual collective persons, created by human convention, are *real* persons in this sense. For *human* real persons, like *all* real persons, are *essentially embodied minds* [21], esp. chs. 1–2 [22]. In turn, the essential embodiment thesis has two logically distinct parts:

(i) the *necessary* embodiment of conscious minds like ours in a living organism (the necessity thesis), and
(ii) the *complete* neurobiological embodiment of conscious minds like ours in all the vital systems, vital organs, and vital processes of our living bodies (the completeness thesis).

The necessity thesis says that necessarily, conscious minds like ours are alive. Negatively formulated, it says that conscious minds like ours cannot be dead, disembodied, or machines. By contrast, the completeness thesis says that conscious minds like ours are fully spread out into our living organismic bodies, necessarily *including* the brain, but also necessarily *not restricted to* the brain. In view of the essential embodiment thesis, specifically human real persons are real persons who are necessarily and completely, *human animals*, hence we are “human, all too human.”

Later in this section, we will work out an explicit metaphysical definition of human real persons. Right now we want to concentrate on the metaphysics of the *dignity* of human real persons as such, or in Kant’s terminology, *Würde*. To say that human real persons have dignity is to say that they’re absolutely, non-denumerably infinitely, intrinsically, and objectively valuable ends-in-themselves. What, more precisely, do we mean by saying that? Objective values are whatever anyone can care about, that is, whatever anyone can aim their emotions (i.e., desires, feelings, or passions) at. Otherwise put, objective values are what Kant called “ends” (*Zwecke*). In turn, “absolute” means “unconditionally necessary.” So to say that human real persons are absolutely, non-denumerably infinitely, intrinsically, objectively valuable ends-in-themselves, or that they have dignity, is to say that their value as ends-in-themelves is not only an unconditionally necessary, internal feature of the kind of manifestly real being they are, but also the very highest kind of value.

Now many things are intrinsically objectively valuable, or ends-in-themselves—for example, pleasant bodily or sensory experiences, vivid emotional experiences, beautiful natural objects and environments, fine craftsmanship, skillfully-played sports, good science, good philosophy, good works of art, and any job well done. To say that human real persons are absolutely, non-denumerably infinitely, intrinsically, objectively valuable ends-in-themselves—i.e., that they have dignity—however, is to say that each of us has a moral value that is a transfinite cardinal quantity in relation to all denumerable or countable, economic, or otherwise instrumental kinds of value, for example psychological pleasure or preference-satisfaction. It seems clear that, no matter how we measure such things, whether in terms of market value or monetary price, degrees of psychological pleasure, degrees of preference-satisfaction, or comparative rankings of such things, nevertheless every actual or possible economic or otherwise instrumental value is expressible as *some rational number quantity or another*, including denumerably infinite rational number quantities. Then, by essentially the same method that Georg Cantor used to show the existence of transfinite numbers [23], at least in principle, we can create a vertical and denumerably infinite list of every actual or possible economic or otherwise instrumental value, then draw a diagonal across it, and discover another value *that is categorically higher than any economic or otherwise instrumental value*. So this value is the prime example of what—following Cantor’s alternative term for transfinite numbers, *transcendental numbers*—we will call *transcendental normativity* [24]. Correspondingly, it is what we will call *transcendental value*, by which we mean either a *single* transcendental value or else a *system* of several distinct but essentially complementary or interlocking transcendental values. Kant also called this *the highest good*. The dignity of human real persons has transcendental value in *that* sense.

Notice too, that even though each human real person has transcendental value—a value that is categorically higher than any economic or otherwise instrumental value and thereby irreducible to any economic or otherwise instrumental value—nevertheless the value of *groups* of human real persons can still be calculated from the cardinality of the membership of the group, just as there is an arithmetic of transfinite cardinal numbers. The dignitarian transcendental value of *N* > 1 human real persons is *N* times greater than the dignitarian transcendental value of one human real person. That is why it is twice as good to save the lives of two human real persons as it is to save the life of one human real person, and twice as bad for two human real persons to die as it is for one human real person to die, and so on. This special kind of transfinite/transcendental value-calculability is true of groups of human real persons, and *yet* the dignity of each human real person has a value that is categorically higher than any economic or instrumental value and thereby irreducible to any economic or otherwise instrumental value, i.e., transcendental value, i.e., the highest good. The highest good in this sense is in each and every one of us; and *that* is the one and only sense in which we are all morally and politically *equal*. But *apart from* that broadly Kantian dignitarian sense of equality, as Harry Frankfurt has compellingly argued [25], *egalitarianism* more generally is a misguided and mistaken moral and political ideal. What is strictly and universally morally and politically obligatory is to treat every human real person *with sufficient respect for their human dignity*, which will *sometimes* involve strict equality of treatment across sets of individual human real persons, but *not* necessarily.

Sufficiently treating a human real person with respect for their human dignity,^1^ in turn, has three individually necessary, individually insufficient, and jointly sufficient conditions: (i) a human real person is sufficiently treated with respect only if they are *not* treated either as a mere means or as mere thing, for example, in the way that Nazis treated people, like a piece of garbage or offal, for no good reason whatsoever, (ii) a human real person is sufficiently treated with respect only if they are treated in such a way that they can give their explicit or implicit rational consent^2^ to that treatment, and (iii) a human real person is sufficiently treated with respect only if they are treated *with kindness*—that is, with benevolent attention to their *true* human needs.^3^ These are mutually logically distinct and individually necessary, but still *individually insufficient* conditions for sufficient respect for human dignity. For, despite what may appear at first glance, they’re not necessarily equivalent, for two reasons.

First, it is at least minimally really possible for a human real person to give their explicit or implicit rational consent to being treated either as a mere means or as a mere thing. Indeed, it is at least minimally really possible that a human real person could explicitly or implicitly rationally consent *even to becoming someone else’s slave or to being killed by that other person*—as an extreme form of self-abasement, self-punishment, self-sacrifice, or sexual self-expression. One real-world example, it seems, is the notorious “German cannibals” case in 2002 [26]. But the more general point we are making here is that in all such cases, someone, of their own free will, *disrespects herself* and therefore is choosing and acting impermissibly, by violating their own human dignity.

In *On Liberty*, Mill famously argued that freely willed self-enslavement is impossible [27], p. 101. But that is a mistake. Freely choosing self-enslavement is really possible. Self-enslavement is putting oneself in bondage, and thus under a system of harsh external restraints, so it is essentially equivalent to self-imprisonment—obviously, an extreme form of putting oneself under a system of harsh external restraints. Both self-enslavement and self-imprisonment are conceptually, metaphysically, and even psychologically coherent, even if, other things being equal, deeply perverse, pragmatically self-stultifying, and morally impermissible. So self-enslavement is not the contrary of freedom. On the contrary, what we call *natural mechanism*, that is, the overwhelming compulsion or manipulation of an agent’s choices or acts by inherently deterministic or indeterministic natural processes, hence metaphysical puppethood or robothood, is the contrary of freedom [16], chs. 1–5]. What is impossible, is to choose freely *while also being* a natural automaton, and this is clearly shown by the soundness of arguments for what is nowadays called *source incompatibilism* [16], Sects. 4.5 and 7.2 [28, 29] in the debate about free will. In rejecting the very ideas of free self-enslavement, Mill confused the concept of *self-stultifying impossibility* with the concept of *freely failing to respect one’s own human dignity*. The latter is obviously immoral, but also obviously *not* impossible, since, just like the *ought*, the *ought-not* also implies *can*.

Second, even if a human real person is *not* being treated as a mere means or as a mere thing, and can *also* give their explicit or implicit rational consent to some proposed mode of treatment, nevertheless she might still be treated *without* kindness. For example, someone who is living in extreme poverty might receive *just enough food aid* not to starve, and *just enough health care aid* not to die from preventable causes, but also *not enough aid* to be well-fed, healthy, self-supporting, or able to engage in any creative, meaningful, useful, or productive activities. Then they are being *oppressed*, by being condemned to a life of constant neediness and suffering.

The upshot, then, is that a human real person is sufficiently treated with respect if and only if (i) they are not being treated either as a mere means or as a mere thing, (ii) they can give their explicit or implicit rational consent to that treatment, and (iii) they are being treated with kindness. In other words, no meaningful act-intention should ever be chosen or acted upon which entails that human real persons are treated either as mere means or as mere things, without their explicit or implicit rational consent, or with cruelty. To treat a human real person without sufficient respect for their human dignity, and thus either as a mere means or as a mere thing, without their explicit or implicit rational consent, or with cruelty, *is to harm them by violating their dignity*. Therefore, it is strictly morally impermissible to harm human real persons by violating their dignity; and for the very same reasons, it is also strictly morally obligatory, to prevent or reduce dignity-violating harms to human real persons. These moral principles are also commonly known as “the negative duty not to harm” and “the positive duty to prevent harm.” Equivalently, a human real person is sufficiently treated with respect for their human dignity if and only if they are provided with *freedom from oppression*.

One direct consequence of this conception of human dignity, which overlaps significantly with early Karl Marx’s political theory, as formulated, for example, in the *Economic and Philosophical Manuscripts* of 1844, is that human real persons are not *commodities* of any kind. Therefore, any person or social institution or system that *commodifies* human real persons, undermines and violates their human dignity.

A second direct consequence of this conception of human dignity, is that human real persons do not have to *do* anything to have dignity, nor can they lose their human dignity *by acting badly*. Human dignity is neither *an achievement* nor *a reward for good conduct*: on the contrary, it is *a constitutive endowment* of their human real personhood.

And a third direct consequence of this conception of human dignity is that it is *not* a general requirement of any human real person’s having dignity that they self-consciously recognize that *they themselves* have dignity, *nor* is it a general requirement of our acknowledging others as having dignity that we self-consciously recognize that *they* have dignity. This is for two reasons.

First, the mental act or state of recognizing oneself or another real person as having dignity is not originally or primarily an act or state of self-conscious, or reflective, report, belief, or judgment. On the contrary, it is originally and primarily an act or state of pre-reflectively conscious emotional perception, or what Michelle Maiese and Robert Hanna have called *affective framing* [21], Sect. 5.3. More precisely, on this view, emotional perception consists in an essentially embodied, conscious, feeling, desiring, passionate intentional agent’s representing the world via* her desire-based readiness to choose or act intentionally*, and, in the midst of that readiness, being disposed *to have feelings about the world, or others, or herself, in certain specific ways*; and the mental content of such acts or states of emotional perception is *essentially non-conceptual* [21], ch. 5 [30, 31], ch. 2]. These same points are also very effectively conveyed by Ludwig Wittgenstein in the *Philosophical Investigations*, without any technical terminology:

“I believe that he is suffering.” –Do I also *believe* that he isn’t an automaton? It would go against the grain to use the word in both connexions. (Or is it like this: I believe that he is suffering, but am certain that he is not an automaton? Nonsense!) Suppose I say of a friend: “He isn’t an automaton.” –What information is conveyed by this, and to whom would it be information? To a *human being* who meets him in ordinary circumstances? What information *could* it give him? (At the very most that this man always behaves like a human being, and not occasionally like a machine.) “I believe that he is not an automaton,” just like that, so far makes no sense. My attitude towards him is an attitude towards a soul. I am not of the *opinion* that he has a soul [32], p. 178e].

Second, the concept of *human dignity*, as we are spelling it out in this essay, is a characteristically *moral-metaphysical* concept that is knowable or known only by rational reflection, moral intuition, and philosophical analysis. It would be paradoxical in the extreme if, for example, someone’s falling deeply in love and regarding another real person as inherently lovable required reflectively knowing the moral-metaphysical analysis of the concept of *love*, either partially or completely. On the contrary, obviously, romantic people normally affectively frame other people as inherently deeply lovable, and thereby fall deeply in love with them, without requiring any reflective or analytical grasp whatsoever of the concepts under which they themselves or the objects of their pre-reflectively conscious emotional perception fall. So too, it would be paradoxical in the extreme if, for example, someone’s either being worthy of respect for their human dignity, or someone’s respecting another human real person, required reflectively knowing the moral-metaphysical analysis of the concept of human dignity, either partially or completely. On the contrary, people normally affectively frame themselves and others as having dignity in a pre-reflective and non-self-consciously conscious way, and without requiring any reflective or analytical grasp whatsoever of the concepts under which they themselves or the objects of their pre-reflectively conscious emotional perception fall.

The metaphysical ground of the dignity of human real persons is their real personhood. And our real personhood is an essentially embodied, unified set of innate cognitive, emotional, and practical capacities. And this is the metaphysical ground of the absolute, non-denumerably infinite, intrinsic, and objective value of all real persons, including of course all human real persons, as ends-in-themselves, precisely because this essentially embodied set of capacities is the only thing in the universe capable of freely recognizing, freely creating, freely acting according to and for the sake of, and freely sustaining, transcendental value or the highest good, which we already know to exist by the Cantorian argument sketched earlier. So human dignity is, at bottom, all about the essentially embodied complex capacity for free will and practical agency that is inherently aimed at transcendental value or the highest good, aka *free agency.* But what, more precisely, is a real person?

Necessarily, every real person is also an individual animal that inherently belongs to some species or another (for example, a *human* real person), but the converse is not the case: not every individual animal within a species is a real person. For example, human infants born with anencephaly—without a cerebrum or a cerebellum, and lacking the top part of the skull—are really *biologically* human, but not human real persons. So not every individual human being is a human real person. Moreover, not every particular living organism within a species is even *an individual animal within that species*, much less a real person in that species. For example, normal human embryos or zygotes prior to 14 days after conception, during the period of “totipotency,” are not even individual human animals, precisely because during that period they can still either split into twins or fuse with several other embryos into a *chimera* [34].

If, necessarily, all real persons are individual animals within some species or another, then obviously we can make some headway towards explicating the nature of real persons only if we are able to answer a preliminary question: “what is an animal?” The Oxford English Dictionary tells us that the word “animal” means “a living organism which feeds on organic matter, usually one with specialized sense organs and nervous system, and able to respond rapidly to stimuli” [34], p. 52. In the usage of contemporary biologists, the term “animal” also has a taxonomical sense, in that animals are said to constitute one of the five kingdoms of living things: Monera (bacteria), Protists, Fungi, Plants, and Animals. The class of animals that is jointly specified by these ordinary language and biological-taxonomical senses includes vertebrates and invertebrates, mammals and non-mammals—including birds, reptiles, amphibians, various kinds of fish, insects, and arachnids. Our usage of the term “animal” throughout this essay, however, is a slight precisification of the ordinary language and biological-taxonomical usages, intended also to coincide with its use in *cognitive ethology*, that is, the scientific study of animal minds and especially non-human animal minds in the context of macrobiology, cognitive psychology, and behavioral psychology [35–39]. To signal this precisification, we have coined the quasi-technical term *minded animal*. Minded animals are living organisms with innate capacities for *consciousness, intentionality*, and *emotion* (including affective sub-capacities for feeling, desiring, and the passions).

Now minded animals are always creatures within some real species *S* or another, hence they are always *S*-type (say, human, or feline, or canine, or equine, etc.) animals. But as we noted above, not every living organism within a species *S* is an individual *S*-type animal. For example, a single human embryo or zygote (that is, the sperm-fertilized ovum) is a living organism within the human species, in the strictly phylogenetic sense of sharing our species-specific biological essence, but a single human embryo is not necessarily a human individual. This is because, as we also noted above, early human embryos up to about the 14^th^ day of their existence are totipotent. This means, among other things, that one embryo can split and later become two distinct human individuals (twins), and also that two embryos can fuse and later become a single human individual (chimeras). What, more generally, is an *individual* belonging to some species *S*, that is, what is an *individual S*-type animal? Our claim is this:

Something *X* is an individual *S*-type (human, feline, etc.) animal if and only if *X* is a living *S*-type organism, and *X* is past the period of totipotency for that species *S*.

Within the human species—and also within a few non-human animal species—many or even most of the animals within that species can also become real persons within that species. The beginning of a real person’s life for a given *S*-type animal is what we call the *neo-personhood* of that animal [17], Sects. 3.1 to 3.3. In the human species, as far as we currently know, the capacity for consciousness first manifests itself in normal fetuses between 25 and 32 weeks after conception or fertilization, hence roughly at the beginning of the third trimester [40], ch. 3. Our view is that this is when your very own human real personal life started—when you became a human neo-person. Prior to that, and from roughly 14 days after your parents conceived the human organism that eventually became you, there also existed a living human animal that also eventually became you—but, just like the totipotent human organism that became that human animal after 14 days, it was not yet you.

This distinction between *animals within a species S* on the one hand, and either *neo-persons* or *actualized real persons within a species S* on the other hand, is a deeply important difference, both metaphysically and morally. This can be seen in at two ways, with specific application to human animals. First, normal human fetuses after the period of totipotency but still before the emergence of consciousness at 25–32 weeks after conception or fertilization, are human animals *but not* human real persons, whether human neo-persons or actualized human real persons. Second, anencephalic human infants—a famous example is the real-world case of Baby Theresa [41], pp. 1–5—are human animals, but neither human neo-persons nor actualized human real persons. Obviously these two claims, if true, will have serious implications for the morality of abortion and infanticide [17], ch. 3.

In any case, every real person is also an *S*-type animal or living organism (but not conversely), and every *individual S*-type animal is also an *S*-type animal or living organism (but not conversely). Therefore, being an *S*-type animal or living organism (although not necessarily an *individual* one, to accommodate totipotent organisms in general and chimeras in particular) is a necessary although not a sufficient condition of real personhood. The rest of our metaphysical analysis of real personhood substantively borrows from two different sources: (i) Harry Frankfurt’s hierarchical-desire theory of persons [42], pp. 11–25, 58–68, 80–94, and 159–176, and (obviously) (ii) Kant’s rationality-based theory of persons in his Critical philosophy.

Frankfurt’s theory of persons is based on the notion of a hierarchically-structured set of desires. The fundamental connection here is that for Frankfurt, a person is essentially *identified with* the constitution of their will, which in turn is a set of desires immanently structured by the capacities for rationality and free agency, and inherently governed by the norm of “decisive identification with effective first-order desires,” that is, by the norm of authenticity or wholeheartedness. In a nutshell, *that* is our view of real persons too, although with a more explicitly and robustly Kantian twist, or rather, set of twists.

But let us now explore some further specific Frankfurtian details, because they are fundamentally important for our broadly Kantian account of real persons. On our broadly Kantian view, a desire is a felt need for something, or a conscious going-for something. This is as opposed to an *actual* need for something—obviously not all felt needs are actual needs—and also as opposed to a mere pro-attitude towards something, a mere preference for something, or a mere wish for something. Frankfurt himself defines the notion of a desire somewhat more broadly, so as to include all pro-attitudes, preferences, and wishes; but in the present context, it is convenient to use our narrower and more conative notion of a desire. Desires in this sense are essentially equivalent with active, committed wants. So to desire *X* is actively and committedly to want *X*; and to desire to *X* is actively and committedly to want to *X*. According to Frankfurt, some animals have not only what he calls *first-order desires*, which are ordinary direct desires for things, events, or real persons (for example, the infant wanting their mother), but also *effective first-order desires*. Effective first-order desires are desires that move (or will move, or would move) the minded animal all the way to action. An effective first-order desire is the same as a minded animal’s *will* or *first-order volition*. First-order desires may or may not be accompanied by *second-order desires*: to want (not) to want *X*, or to want (not) to want to *X*. If so, then some of the second-order desires may be directed to the determination of precisely which first-order desire is to be the effective first-order desire, that is, the minded animal’s will and first-order volition; and such desires are *second-order volitions*.

According to Frankfurt, whatever the order-level of desires or volitions, they can be either conscious or non-conscious. For the purposes of our discussion, however, we will concentrate exclusively on *conscious* desires and volitions. This is, in part, because we think that there is no such thing as a mental state, whether dispositional or occurrent, that is strictly non-conscious and not to some non-trivial degree occurrently conscious. In earlier work, Maiese and Hanna have called this (admittedly controversial, but also, we believe, defensible) claim “The Deep Consciousness Thesis” [21], chs. 1–2, [22]. But in any case, and according to Frankfurt, all and only persons have second-order volitions, because all and only persons care about the precise constitution of their wills. By contrast to persons, creatures that are “wantons” have effective first-order desires, but they either lack second-order desires (hence they cannot care about the precise constitution of their wills because they lack self-conscious desires) or if they have second-order desires they nevertheless lack second-order volitions (hence even though they have self-conscious desires, they still cannot care about the precise constitution of their wills). Again, according to Frankfurt, all non-human animals, all human infants, and some human adults are wantons. Finally, for Frankfurt a person has freedom of the will if and only if they can determine, by means of a second-order volition, precisely which among their first-order desires is the effective one. This is also known as *identification* or *decisive identification* [42], pp. 58–68, and 159–176; otherwise persons have unfreedom of the will. Wantons have neither freedom of the will nor unfreedom of the will, simply because they are not persons.

We accept much of what Frankfurt has to say about persons and their wills, and correspondingly we want to apply much of what he says to *human real* persons and *their* wills. Nevertheless, we also have substantive disagreements with him on two mid-sized (as opposed to either major or minor) points.

Our first mid-sized point of substantive disagreement is that we doubt that Frankfurt’s notion of personhood adequately captures the full breadth or depth of our broadly Kantian notion of human real personhood, according to which some human real persons have what we will call *higher-level or Kantian rationality*. This, in turn, is an innate complex capacity for strict-norm-guided logical or practical reasoning, for reflective self-consciousness, for autonomy or self-legislation, for authenticity or wholeheartedness, and for moral or non-moral responsibility. Any minded animal that also has higher-level or Kantian rationality can recognize necessary truths, judge or believe with a priori certainty, and choose or act wholeheartedly in accordance with desire-overriding non-instrumental, non-selfish, non-egoistic or non-self-interested, non-hedonistic, non-consequentialist, categorically normative reasons and duties, that is, those reasons and duties that inherently express the Categorical Imperative and the “categorical ‘ought’.”

By sharp contrast, what we will call *lower-level or Humean rationality* involves only the possession of innate capacities for conscious, intentional desire-based logical or practical reasoning, for more or less momentary or occasional occurrent self-consciousness, and for self-interested, or in any case instrumental, intentional agency. Any minded animal that has lower-level rationality can recognize contingent truths, judge or believe with a posteriori certainty, and choose or act in accordance with broadly instrumental egoistic, hedonistic, or consequentialist reasons and duties, or those that express at most the “hypothetical ‘ought’.”

All minded animals that possess an innate capacity for higher-level or Kantian rationality also possess an innate capacity for lower-level or Humean rationality, but not the converse. For example, it is arguable that normal, healthy Great apes and perhaps also dolphins^4^ possess an innate capacity for Humean or lower-level rationality, but not a capacity for higher-level or Kantian rationality. This is of course *not* to say that Great apes or dolphins are “irrational” or “non-rational” in any sense. On the contrary, it is only to say that, relative to those animals that *do* possess an innate capacity for higher-level or Kantian rationality, the rational capacity of Great apes and perhaps also dolphins is somewhat limited in complexity and normative power. Minded animals with an capacity for rationality in the higher-level or Kantian sense are not only constrained in their intentional agency by the Categorical Imperative or at least by some strictly universal, non-instrumental, altruistic, non-hedonistic, and non-consequentialist moral reasons and objective principles, they are also capable of being moved wholeheartedly by the moral emotion of broadly Kantian respect for dignity. Or in other words, minded animals with a fully online capacity for rationality in the higher-level or Kantian sense are also capable of broadly Kantian *autonomy* and what we call *principled authenticity* [16], esp. chs. 3, 5, and 6 [17], esp. chs. 1 and 6.

By contrast, minded animals that possess only an innate capacity for rationality in the lower-level or Humean sense are constrained in their intentional agency only by (at least some of) the axioms of rational choice theory, but not by strictly universal, non-instrumental, altruistic, non-hedonistic, and non-consequentialist moral reasons and objective principles. They are therefore not capable of broadly Kantian autonomy or principled authenticity. Instead, they are at most capable of being moved non-authentically or non-wholeheartedly by the first-order Humean moral emotion of *sympathy* [46], books II and III.^5^

What is the moral-emotional difference between broadly Kantian respect for human dignity and Humean sympathy? One way of cashing out this difference is to say that whereas (i) someone who is being moved by broadly Kantian sufficient respect for human dignity will always and necessarily choose and act so as to heed or preserve the dignity of another human real person, even if she does not find that other human real person to be *in any way whatsoever* attractive, likeable, nice, tear-jerkingly pathetic, or pleasant—in short, even if they involuntarily find that human real person to be perfectly loathsome, nevertheless (ii) someone who is being moved merely by Humean sympathy will choose and act so as to heed or preserve the dignity of another human real person *only if* they  find that human real person to be appropriately attractive, likeable, nice, tear-jerkingly pathetic, or pleasant.

Some human animals are “persons” in Frankfurt’s sense, hence are human real persons in our sense, and also rational agents in the lower-level or Humean sense, and not rational agents in the higher-level or Kantian sense, but this is *not* because they lack the innate capacities for agency in the higher-level or Kantian sense. Rather they *do* possess these capacities, but in the mode of *real potentiality that is not yet actualized*, hence it it is simply because they are *not-yet* rational agents in the higher-level or Kantian sense. Indeed we—the actual and really possible readers of this essay—were all of us, for a time, such creatures.

Thus human real personhood in the lower-level, Frankfurtian sense is a necessary and sufficient condition of human real personhood, which includes all the more-or-less online basic capacities of free agents, hence it entails human dignity. And as we have seen, it is based on the fully online capacity for having second-order volitions, which in turn contains several other distinct constituent fully online psychological capacities. Human real personhood in the higher-level, Kantian sense, i.e., moral agency, on the other hand, both includes and significantly augments human real personhood in the Frankfurtian sense, by including the fully online capacity for principled authenticity, at least partially or to some degree. Correspondingly, human real personhood in the higher-level, Kantian sense is based on the fully online capacity for higher-level rational agency, which also contains several other distinct online psychological capacities. To display the internal complexity of the relationships between these capacities more fully, here is an explicit version of the two-level theory of human real personhood that we have been developing, together with our conception of human neo-personhood, in the form of a four-part metaphysical definition of human real personhood:

**Part I.** *X* is a *Frankfurtian human real person* (person_f_) if and only if *X* is a human animal and *X* has fully online psychological capacities for

(i) essentially embodied consciousness or essentially embodied subjective experience,

(ii) intentionality or directedness to objects, locations, events (including actions), other minded animals, or oneself, including cognition (that is, sense perception, memory, imagination, and conceptualization), and caring (that is, affect, desire, and emotion), especially including effective first-order desires,

(iii) lower-level of Humean rationality, that is, logical reasoning (including judgment and belief) and instrumental decision-making,

(iv) self-directed or other-directed evaluative emotions (for example, love, hate, fear, shame, guilt, pride, etc.),

(v) minimal linguistic understanding, that is, either inner or overt expression and communication in any simple or complex sign system or natural language, including ASL, etc., and.

(vi) second-order volitions.

**Part II.** *X* is a *Kantian human real person* (person_k_), aka *a human moral agent*, if and only if *X* is a human real person_f_ and also has fully online psychological capacities for

(vii) higher-level or Kantian rationality, that is, categorically normative logical rationality [47], esp. chs. 6–7 and practical rationality, the latter of which also entails a fully online capacity for deep (non-)moral responsibility, autonomy (self-legislation), and wholeheartedness, hence a fully online capacity for principled authenticity, at least partially or to some degree.

**Part III.** *X* is a *human real person* if and only if *X* is either a human real person_f_ or a human real person_k_; and any other finite, material creature or entity *X* is a non-person.

**Part IV.** If *X* is an actualized human real person, then *the neo-person* of *X* is also a human real person, where the neo-person of *X* is an individual human animal *A* that manifests the psychological capacity for consciousness and the following counterfactual is also true of *A*:

If *A* were to continue the natural course of its neurobiological and psychological development, then *A* would become *X*.

## Human happiness or flourishing, true human needs, and human dignity

All human persons have a fundamental need for happiness, aka *flourishing*, and happiness or flourishing consists in the satisfaction of what we call *true human needs*.^6^ Some true human needs are such that their active satisfaction is a necessary condition of all human dignity. We will call those *the lower-level basic human needs.* For example, among the lower-level true human needs are everyone’s needs for (i) adequate nourishment, adequate clothing, and adequate accommodation (provision), (ii) adequate physical and mental health, as sustained by adequate healthcare, (iii) adequate access to a healthy natural environment, both local and global, (iv) adequate scope for human movement and travel across the earth, (v) adequate protection from coercion by others (safety), (vi) adequate access to human companionship and human communication, and (vii) adequate primary and secondary education for the development and exercise of the innate capacities that collectively constitute personhood. By “adequate” in each case, we mean *sufficient, in view of all relevant empirically well-supported tests that also fully conform to basic moral principles of human dignity*. Since satisfying these true human needs is a necessary condition for human dignity, then sufficient respect for human dignity demands that everyone, everywhere should always have enough of whatever it takes to satisfy their lower-level true human needs.

Over and above the lower-level true human needs, all other true human needs are those whose satisfaction most fully conform to the absolute, non-denumerably infinite, intrinsic, objective value of human dignity. Indeed, they are *humanity-realizing needs*. More precisely, the satisfaction of such needs allows people to activate and to exercise their various capacities and realize their potentiality for being autonomous, individually flourishing, and collectively flourishing, in ways that also are fully compatible with and fully supportive of the agential autonomy, relational autonomy, individual flourishing, and collective flourishing *of everyone else*. Let us call these *the higher-level true human needs*, since they presuppose the satisfaction of lower-level basic human needs, and also most fully realize the innate capacities of our personhood. For example, among the higher-level basic needs are everyone’s needs for (i) aesthetic enjoyment of all kinds, (ii) personal relationships of all kinds, for example, families, life-partners, lovers, close friends, a wider circle of friends, comrades, etc., (iii) social and political solidarity of all kinds, (iv) free thought and free speech of all kinds, (v) creative self-expression of all kinds, (vi) meaningful work of all kinds, (vii) higher education of all kinds, and (vii) spirituality of all kinds. Since it is arguable that the ultimate goal, purpose, or meaning of human life is no more and no less than to pursue the satisfaction of higher-level true human needs, then sufficient respect for human dignity also demands that everyone, everywhere, should always have enough of whatever it takes for them to be able to pursue their higher-level true human needs [9], ch 3.

## Our moral obligations with respect to all human real persons

It is morally obligatory for everyone to treat all human real persons, including ourselves, with sufficient respect for their/our dignity, which in turn entails (i) never treating human real persons as *mere means* to our own goals, and especially never treating them as *mere things* (especially via coercion), (ii) always treating human real persons consistently and impartially (except insofar as an expression of partiality—say, towards one’s own family members—also sufficiently respects their dignity as well as the dignity of others), (iii) always treating human real persons in a way that makes it possible for them to be happy and therefore to satisfy their true human needs, (iv) never treating human real persons in such a way as to undermine their capacity for free agency, and (v) always treating human real persons as belonging to a universal, worldwide community of human real persons who mutually sufficiently respect one another’s dignity. Any violation of any of the five moral obligations just formulated, whether it is aimed by a given human real person at another individual or group of individuals specifically, or whether it is built into the very structure of a social institution, is what we will call *oppression*—and more specifically, we will call the former kind of oppression *individual oppression*, and the latter kind of oppression *systemic oppression*.

## What a natural automaton or natural machine is

Anything *X* is a *natural automaton,* or *natural machine*, if and only if

- (i) *X* is constituted by an ordered set of causally efficacious behaviors, functions, and operations (aka “causal powers”),
- (ii) the causal powers of *X* are necessarily determined by all the settled quantity-of-matter-and/or-energy facts about the past, especially including The Big Bang, together with all the general deterministic or indeterministic causal laws of nature, especially including the Conservation Laws, and.
- (iii) *X*’s causal powers are all inherently effectively decidable, recursive, or Turing-computable, given two further plausible assumptions to the effect that (iiia) the causal powers of any real-world Turing machine are held fixed under our general causal laws of nature, and (iiib) the “digits” over which the real-world Turing machine computes constitute a complete set of mathematically denumerable (that is, non-real-number, non-complex-number, non-transfinite) quantities, that is, spatiotemporally discrete, physical objects.

## Why human real persons are not natural automata/natural machines: because consciousness is a form of life

According to what we will call *the mechanistic worldview*, everything whatsoever in the world, including all human activity, is fully and ultimately explicable by mechanical principles alone (including principles of computability and/or mathematical physics, including chemistry, and biology insofar as it is reducible to physics and chemistry). In turn, and more specifically, the mechanistic worldview consists in the conjunction of three somewhat distinct but logically nested theses:

- (i) *formal mechanism*, applied to mathematics, logic, truth, and knowledge more generally, namely the theory of computability and recursive functions, including decidability [12, 13],
- (ii) *natural mechanism*, which applies the notion of a natural automaton or natural machine, as per Sect. 7immediately above, to everything in the material or physical world [16], esp. ch. 2 [48], and.
- (iii) *scientific naturalism*, applied to everything in the world, including all human activity, which includes formal and natural mechanism, scientism (i.e., the valorization of the formal and/or natural sciences and their methods), empiricism, and materialist/ physicalist metaphysics (i.e., everything in the world is either identical to or necessarily dependent on fundamentally physical contingent facts) [49, 50].

Sharply on the contrary, in our opinion, to understand the nature of conscious mind in general and rational human conscious mind in particular, we need radically to re-think our *concept of nature* itself, radically re-conceiving nature as inherently *processual and purposive*, running from The Big Bang Singularity forward, via temporally asymmetric or unidirectional energy flows, to organismic life, and then on to conscious mind in general and to rational human conscious mind in particular, which in turn entails including radically re-conceiving the mind–body relation, free agency, and emergence. In a nutshell, our thesis is *that there is a single, unbroken metaphysical continuity between The Big Bang Singularity, temporally-asymmetric/unidirectional energy flows, organismic life, and conscious mind* [51]. For convenience and simplicity’s sake, we will call this the *conscious-mind-is-a-form-of-life* thesis, aka *the CMFL thesis*.

The metaphysics of the mind–body relation that directly answers to the CMFL thesis is that the mental-physical relation is a *two-way necessary complementarity*, that is, a mental-to-physical and physical-to-mental necessary equivalence that captures the manifestly real essence of minded animals like us. In short, as we mentioned in Sect. 4 above, and as Michelle Maiese and Robert Hanna put it, minded animals like us are *essentially embodied minds*: hence we call this “the essential embodiment theory,” or EET [21].

In a nutshell, EET says that the conscious minds of animals are *necessarily* and *completely* embodied in those animals, and, more specifically, that the conscious mind of an animal is the global dynamic immanent structure of the living organismic body of that very animal, a structure that inherently activates and guides the animal’s causally efficacious biological powers—or as Aristotle puts it in his own terminology: “the soul (*anima*) is the first actuality of a natural body that has life potentially” (*De Anima*, II.i.412a22). Hence EET is committed to a dynamicist, organicist, and processualist version of *neo-Aristotelian hylomorphism* about the mind–body relation [21], esp. chs. 1–2 and 6–8.

Consciousness, in turn, is *subjective experience*, which is to say that it inherently involves a self that’s egocentrically-centered in orientable space and asymmetric/unidirectional time (= subjectivity), and also that this self enacts or engages in mental acts, states, or processes of various kinds (= experience), and furthermore consciousness has two basic modes: (i) *pre-reflective or non-self-conscious consciousness*, which, in being naturally directed towards cognitive or intentional targets other than itself, is immanently reflexive, or aware of itself egocentrically and internally, without implicitly or explicitly forming judgments or propositional thoughts about itself, and (ii) *reflective consciousness*, or *self-consciousness*, which, in being naturally directed towards, or about*,* itself AS a cognitive or intentional target, is transcendently reflexive, or aware of itself allocentrically and externally*,* by implicitly or explicitly forming judgments or propositional thoughts about itself. More simply put, pre-reflective or non-self-consciousness consciousness is just *being* a conscious mind that is directed towards other animals or things; whereas reflective or self-conscious consciousness is thinking about itself AS a conscious mind that is ALSO directed towards other animals or things.

EET is a specially restricted version of “dual-aspectism*.*” For examples of other dual aspect theories, one can compare and contrast Spinoza’s theological monism (in *The Ethics*), Bertrand Russell’s neutral monism (in *The Analysis of Mind* and *The Analysis of Matter*), or A.N. Whitehead’s universal panexperientialist organicism (in *Process and Reality*). Unlike Whitehead’s universal panexperientialist organicism, however, EET does not say that *everything, everywhere in the world* is somehow minded, as an intrinsic nonrelational property of that thing, from the fundamental level up. For that would mean, for example, that even Dale’s Pale Ale and the cans that contain it are somehow minded, as an intrinsic nonrelational properties of those things, which is clearly an excessively strong metaphysical thesis. Nevertheless EET does, in a specially restricted way, share some of the metaphysical benefits of panpsychism—namely, that in all and only suitably complex kinds of organismic living creatures and their life-processes, causally efficacious mental and physical properties are related by two-way necessary complementarity. Or in other words: all and only everything in the world that is the right kind of organismic living creature and its life-process, is minded. So EET is a specially restricted version of psycho-organicism.

More specifically, however, EET says (i) that minds like ours are necessarily and completely embodied, (ii) that minds like ours are complex global dynamic structures of our living organismic bodies, i.e., *forms of life*, (iii) that minds like ours are therefore inherently alive, (iv) that minds like ours are therefore inherently causally efficacious, just like all forms of organismic life, and (v) that minds like ours emerge over time and in space in all and only certain kinds of living organisms, i.e., minded animals.

Furthermore, if by *autonomy* we mean *a capacity for self-determination in the broadest possible sense*, then we can also distinguish between (v1) *the autonomy of proto-consciousness*, a minimal and relatively self-less endogenous sensibility possessed by all living organisms, all the way down to unicellular organisms, (v2) *the autonomy of pre-reflective consciousness*, an egocentric and immanently self-aware, self-locating sensibility possessed by all minded animals, and (v3) *the autonomy of self-consciousness*, a further and specifically rational conscious capacity to represent oneself by means of concepts and judgments, which requires and indeed presupposes that we are also able to think propositionally, speak richly-structured natural languages, and engage in logical reasoning [47], ch. 4.

Now in addition to self-consciousness, obviously rational human minded animals like us are also inherently capable of (i) consciousness, that is, subjective experience (as defined above), but also (ii) *intentionality*, that is, directedness to all kinds of things as their cognitive, desiderative, emotional, etc., targets. These capacities for consciousness and intentionality are also shared with minded animals in many other species, but *self-evidently* manifest themselves in minds like ours, via our further capacity for specifically *rational* consciousness, intentionality, and self-consciousness, not only as per Descartes’s *Cogito*, “I think, therefore I am,” but also, and even more fundamentally, via our capacity for essentially embodied *emotional* consciousness, intentionality, and self-consciousness, as per what Maiese and Hanna call *the Essentially Embodied Cogito*, “I desire, therefore I am.” [21], p. 21.

In any case, the two fundamental problems in classical philosophy of mind are these:

*The mind–body problem*: what accounts for the existence and specific character of conscious, intentional minds like ours in a physical world?

*The problem of mental causation*: what accounts for the causal efficacy and causal relevance of conscious, intentional minds like ours in a physical world?

Correspondingly, here are eight reasons why EET, when foregrounded against the backdrop of the CMFL thesis of a single, unbroken metaphysical continuity running from The Big Bang Singularity, via temporally asymmetric/unidirectional energy flows, to organismic life, to conscious, intentional minded animals, to self-conscious rational human minded animals like us, not only *dissolves* the classical mind–body problem and the problem of mental causation, but also finally *solves* them, in the sense that the CMFL + EET combination presents a new and arguably true view of the mind–body relation against the backdrop of a radically revised conception of nature.

First, EET fully avoids *reducing* the mental to the physical, aka *reductive physicalism*. Reductive physicalism, presenting itself via the sheep’s clothing of the mind–body identity theory or the logical supervenience of the mental on the physical, de facto simply *eliminates* the mental. But what could be more epistemically primitive than our subjective experience of ourselves *as* conscious, intentional minds, and correspondingly, what then could be more metaphysically and ontologically primitive than the fact of the mental *quâ* mental?

Second, EET fully avoids making the mental naturally or nomologically supervenient on the physical, aka *non-reductive physicalism*. Reductive physicalism entails epiphenomenalism, hence it robs the mental of all its efficacious causal power. It is no solution to say that, from a non-reductive physicalist point of view, the mental can still have “causal relevance”: on the contrary, the mental has got to have *efficacious causal powers*, not merely an important informational bearing on causal processes.

Third, EET fully avoids reducing the physical to the mental, aka *subjective idealism*. Subjective idealism makes nature’s existence radically dependent on the existence of individual minds. It is highly implausible to hold that physical nature came into existence *only after* there were any minded animals. For, since animals are parts of physical nature, it would follow that animals came into existence only after there were minded animals. And it is equally highly implausible to hold that if all individual minds were to perish, physical nature would go out of existence too. For in that case, since all animals die, and in most cases after animals die, their corpses continue to exist for a while, it would follow that necessarily, the last minded animal would have no corpse.

Fourth, EET fully avoids making the mental and the physical either essentially or even logically independent of one another, as per either Cartesian “interactionist substance dualism” or Cartesian “property dualism.” Any form of Cartesian dualism makes it impossible to explain how the mental and the physical causally interact without appealing to some sort of metaphysical mystery: for example, Descartes’s God, Leibniz’s divine pre-established harmony, an ectoplasmic medium, etc., etc. And any form of Cartesian dualism also entails the metaphysical impossibility that subjective experiences could exist without embodiment.

Fifth, EET fully avoids over-restricting mentality to the brain, i.e., it fully avoids the error of “the brain-bounded mind” [22].

Sixth, EET fully avoids *over*-extending the mental beyond the living animal body, i.e., it avoids the error of “the extended mind” [52, 53].

Seventh, EET provides adequate metaphysical foundations for a robust metaphysics of free agency [16].

Eighth, and perhaps most importantly, building on the sixth and seventh points, EET is an approach to the mind–body problem, including the problem of mental causation, which is perfectly scaled to the nature, scope, and limits of our “human, all too human” existence in a thoroughly non-ideal natural and social world. Brain-boundedness falls short of the human condition: it makes us much less than we manifestly really are. The extended mind exceeds the human condition: it makes us much more than we manifestly really are. Only the essential embodiment of the mind adequately captures and reflects the human condition: it tells us exactly what we manifestly really are. For every human real person just *is* their minded animal body and its “human, all too human” life, for better or worse. In short, EET answers perfectly to Socrates’s Delphic-Oracle-inspired thesis that an ultimate aim of philosophy is to “know thyself.”

Now back to the the mechanistic worldview. It receives a paradigmatic application in the use of psychometrics and data in digital technology and computers, since algorithmic nudging is premised on the assumption that human animals are natural automata/natural machines. Correspondingly, against the backdrop of the CMFL thesis and EET, here are seven arguments against the mechanistic worldview. Assuming the soundness of the arguments, one crucial further implication of the seven arguments taken collectively is to put in serious question the readiness with which formal and/or natural mechanism are typically *assumed* in psychometrics. More generally, we present these arguments for two reasons: (i) to show that are many distinct sorts of challenges to the mechanistic worldview, and, correspondingly, (ii) to shift the burden of proof: usually, anti-mechanists (aka *organicists*) are assumed to be fighting a purely defensive philosophical battle against a *prima facie* “self-evident” mechanistic worldview, whereas what we seek to show is that in reality what is *prima facie* self-evident are human personhood and organicism, and that the mechanistic position is in reality in a purely defensive position [54, 55]. We have also grouped our arguments into three clusters: (A) logical and mathematical arguments (arguments 8.1 and 8.2), (B) physical and metaphysical arguments (arguments 8.3 and 8.4), and (C) mentalistic and agential arguments (arguments 8.5, 8.6, and 8.7).

**(A) Logical and mathematical arguments**

**8.1 From “the logocentric predicament”**

The logocentric predicament says that in order to explain or justify logic, (a minimal classical proto-) logic must be presupposed and used, but every explanation whatsoever presupposes and uses (a minimal classical proto-) logic, including all mechanistic explanation, hence (a minimal classical proto-) logic cannot itself be mechanically explained [47], ch. 1. In other words, every explanation and justification of logic is *circular*, since it already assumes and deploys logic, but this circularity is not rationally *vicious*: on the contrary, it is rationally *virtuous*, since what it shows is that logic is more fundamental than and irreducible to *any* kind of explanation, especially including *mechanistic* explanation [47], ch. 3.

**8.2 From the incompleteness of mathematics and mathematical logic**

Kurt Gödel proved in 1931 that there are some uncomputable/undecidable, unprovable true sentences in Peano arithmetic plus *Principia-Mathematica* style formal logic, and that truth-determination and consistency in any logico-mathematical system rich enough to include Peano arithmetic and *Principia*-style logic must occur outside that logico-mathematical system itself, yet by hypothesis these sentences are indeed true, and also we can know them to be true (say, by mathematical intuition), hence (i) truth-determination and consistency in any logico-mathematical system rich enough to include Peano plus *Principia*-style formal logic cannot be mechanized, and (ii) our mathematico-logical knowledge cannot be mechanized [56,57, 58].

**(B) Physical and metaphysical arguments**

**8.3 From the incompleteness of mathematical physics**

(8.3i) Mathematical physics presupposes mathematics and mathematical logic, so because mathematics and mathematical logic are formally incomplete by Gödel’s theorems, then so is mathematical physics, and therefore formal truth and knowledge in mathematical physics cannot be mechanized.

(8.3ii) Quantum uncertainty and indeterminacy show that certain micro-physical events cannot be predicted by the Standard Models of cosmology and particle physics (for example, which way a single particle will go in the Two-Slit Experiment, etc.), yet many essentially analogous macro-physical events involving human free agency can be reliably predicted by the agents themselves, so mathematical physics as per the Standard Models is empirically incomplete, and therefore not only formal truth and knowledge, but also *empirical truth and knowledge*, in mathematical physics cannot be mechanized [55], Sect. 15.

**8.4 From the irreducibility of biology to physics**

Living organisms cannot be fully explained according to the physical laws and principles governing naturally mechanistic systems [51]. The crucial move here is to recognize that there are essential homologies between the atom and the unicell that provide a unifying explanatory common denominator between them, thereby establishing a three-step irreducible metaphysical continuity between (i) non-equilibrium thermodynamic temporally asymmetric/unidirectional energy flows constituting the classical micro-physical quantum-theoretic particle/wave duality, (ii) organismic life per se, and (iii) minded animal organismic life.

**(C) Mentalistic and agential arguments**

**8.5 From consciousness (i.e., subjective or “lived” experience) and objective or “directed” experience (aka “intentionality”)**

The specific characters (or qualities) of human consciousness and directed experience/intentionality can vary independently of any and all physical facts and properties, including functional facts and properties, therefore metaphysical materialism or physicalism is false: see, for example, (i) the “Chinese Room Argument” (someone inside a room who successfully manipulates Chinese inputs and outputs, thereby passing the Turing test, can consciously realize that they do not understand Chinese) [59], (ii) the “Zombie Argument” (there are conceivably possible physical counterparts of a conscious animal, that lack consciousness) [60], chs. 1–8, and above all, (iii) the “Necker Cube Argument” from multistable perception of, for example, the Necker Cube (there are conceivably possible distinct mirror-reflected/enantiomorphic perceptual aspect-counterparts that can be paired with the same physical states, and physical causation alone fails to determine precisely which mirror-reflected aspect of the Necker Cube will be paired with that same physical state) [21], p. 281 [31], pp. 94–97.

**8.6 From intrinsic motivation**

Whether artificial or natural, machines cannot be intrinsically motivated to choose or do *X*, only extrinsically caused or programmed for bringing about *X*, yet we can freely choose or do many different kinds of things for their own sake (see Sect. 4 above), and when this capacity for free agency is conjoined with the CMFL thesis and EET, it follows that our intrinsic motivation cannot be mechanized.

**8.7 From transcendental motivation**

Whether they are artificial or natural, machines cannot choose or do *X* for the sake of transcendental value, aka the highest good, precisely because they inherently lack consciousness (as per the CMFL thesis and EET) and free agency [16], esp. chs. 1–5, and are only extrinsically caused or programmed for bringing about *X*, yet we can freely choose and do many different kinds of things precisely because they are *neither* egoistic or self-interested (private utility) *nor* (merely) beneficial for everyone else (public utility), but simply for the sake of transcendental value/the highest good, e.g., acting for the sake of sufficient respect for human dignity (see Sect. 4 above), hence our transcendental motivation cannot be mechanized.

Therefore, in view of the the broadly Kantian theory of human dignity, the CMFL thesis, EET, and these seven arguments, human real persons are *not* natural automata/natural machines, because, necessarily, every human real person is a living organism inherently possessing a certain set of innate capacities, especially including consciousness and free agency, with dignity, and no natural automaton/natural machine is either a living organism or innately possessed of these capacities [51, 55], or has dignity.

## Our moral obligations with respect to the design and use of artificial automata or artificial machines, aka computers, and digital technology more generally

It is morally impermissible to design and/or use any artificial automaton or artificial machine—any computer—and digital technology more generally, in such a way as to fall short of or more generally violate sufficient respect for the human dignity of human real persons. This covers an extremely wide variety of impermissible designs and/or uses, including: digital manipulation of human real persons via “nudging” strategies specifically designed to undermine the satisfaction of true human needs; digital demagoguery; digital coercion of human real persons; digital threats of violence or other significant non-violent harm directed at human real persons more generally; digital slander of individual human real persons; active digital discrimination against real persons of any kind whatsoever; algorithms that prevent people from gaining access to digitally-stored information that is necessary for being treated with sufficient respect for their human dignity, especially including the satisfaction of true human needs, or is required for their self-protection against, protection of others against, or resistance to, any sort of individual oppression or systemic oppression; and legally permitted ubiquitous data collection without rational consent (more on this in Sect. 10).

In this connection, it is extremely important to note that freedom of speech and freedom of expression more generally play a special moral role as humanity-realizing needs or higher-level basic human needs, in that when they are properly practiced, they *articulate and disseminate* our core dignitarian commitments and humanistic values. But if, in pursuit of this dignitarian aim, someone’s digitally-expressed or digitally-stored free speech or other form of self-expression *merely offends* another person and/or draws the ire of those who administer or govern coercive authoritarian social institutions, then this does *not* entail a morally impermissible design and/or use of computers or digital technology more generally. Our merely offending someone and/or annoying adminstrators or governments, is *not* the same as our oppressing others, whether individually or systemically.

The revelant distinction here is between (i) any human real person’s being treated in such a way that this mode of treatment fails to have sufficient respect for their dignity or even outright violates their dignity, which is *being oppressed*, and (ii) any human real person’s merely being greatly annoyed by something that someone else says or writes, or by some other human real person’s attitudes or beliefs, even though it does *not* involve actual oppression, which is *being offended*. Being oppressed, therefore, is of real moral significance, whereas being offended is *morally insignificant*, even if it is of real psychological significance for the human real person who is offended by someone else’s speech, attitudes, or beliefs.

## What privacy is, why invasions of digital privacy are morally impermissible, whereas consensual entrances into digital privacy are either morally permissible or even obligatory

To be sure, privacy is not the be-all and the end-all of digital ethics/AI ethics; nevertheless it *is* important, especially in view of the rise and widespread use of digital technology for legally permitted ubiquitous data collection—for example, as applied to distance workers during the 2020–2021 COVID-19 pandemic [61].

Now *privacy* is the sphere bounded by the living animal body and by the entire life of an individual human person, including all information about that person that uniquely identifies them, and especially all digitally-stored information that uniquely identifies them; for example, digitally-stored medical information, digitally-stored biophysical information more generally, digitally-stored photographs or videos of that person, digitally-stored financial information, and digitally-stored social information more generally, especially including that person’s use of computers and/or other digital technology for surfing the internet and using social media. In view of our moral obligations with respect to all human real persons, no one can morally permissibly enter into the privacy of another human real person without their explicit or implicit rational consent, and in particular, no one can morally permissibly enter into the *digital privacy* of another person without their explicit or implicit rational consent. Or in other words, rational consent is what freely opens the door into the sphere of privacy, whether this is non-digital or digital. The occurrence of such an entrance without rational consent—an uninvited foot in the door, slipping inside while no one is watching, or breaking down the door—is what we will call *an invasion of privacy*, and in cases of digitally-stored information, *an invasion of digital privacy*. Invasions of digital privacy are violations of sufficient respect for human dignity, and therefore morally impermissible.

It is also extremely important to note that explicit or implicit rational consent can be given for the use of uniquely identifying information. Therefore, not every entrance into privacy is morally impermissible. Indeed, a great many entrances into privacy are not only consistent with but even morally obligatory to secure sufficient respect for human dignity, and fully involve actual or implicit rational consent: hence we will call them *consensual entrances into privacy*. In particular, there can be *consensual entrances into digital privacy*. For example, if a doctor uses digitally-stored uniquely identifying medical information about a certain human real person, a use that has been actually or implicitly rationally consented to by that human real person, to save that human real person’s life, then this entrance into digital privacy sufficiently respects that person’s dignity, and at the very least it is morally permissible, and perhaps even (say, in view of the doctor’s Hippocratic Oath) morally obligatory.

What is the criterion of *implicit rational consent*? The basic idea is that if any human real person *were*, by means of a thought-experiment, placed behind what John Rawls called a “veil of ignorance,” [62] which procedurally screens out all uniquely self-identifying personal identity details from that person’s own cognitive and practical point of view, and temporarily ensures a suitable reflective disinterestedness and distance from their actual “human, all too human” condition, then they would agree to that treatment. The notion of implicit rational consent is important in cases for which the real-world moral context is so “messy” that the human real person has little or no opportunity to reflect and make a well-considered judgment; for which the human real person has, at that time, insufficient knowledge of digital technology and/or computers; for which there is good reason to believe that the human real person is not psychologically competent in that particular context; for which there is good reason to believe that the human real person is being coerced in that particular context; or for which there is good reason to believe that the human real person is under some or another serious cognitive illusion in that particular context. Since a great many real-world moral contexts are such that explicit rational consent or its refusal is simply out of the question, the notion of implicit rational consent or its refusal plays an essential role in digital privacy.

Now applying this set of ideas to legally permitted ubiquitous data collection, this is clearly an invasion of digital privacy—it is clearly one thing to evaluate workers’ performances, via all-things-considered judgments, but sharply another to spy on them—and therefore morally impermissible, unless the workers explicitly or implicitly rationally consent to this treatment. And it would also be morally impermissible coercion on the part of bosses and management to require workers to agree to this ubiquitous data collection as a necessary condition of accepting job offers or of keeping their jobs. So, *yes it is* “an Orwellian Big Brother arrangement,” [61] *even if* it is legal. And this in turn raises one final important general issue in the area of digital ethics/AI ethics, namely, a basic distinction between dignitarian morality and legality.

## Dignitarian morality versus legality, and digital ethics/ai ethics

Morality, as we have defined it for the purposes of this essay, is the attempt to guide human conduct by rationally formulating and following principles or rules that reflect our basic personal and social commitments and our leading ideals and values. But by contrast, legality concerns what is deemed right or wrong by social institutions (for example, business corporations, universities, governments, judicial systems, etc.) that enforce their judgments, policies, rules, and laws by means of coercion.

But, contrary to an influential trend in moral philosophy, jurisprudence, and political philosophy since the late 1970s [63], human social institutions institutions (say, business corporations, judicial systems, the police, governments, etc.) are *not* human real persons, as our definition of human real personhood clearly shows (see Sect. 4 above), and therefore no matter how coercively powerful they might be, social institutions have no human dignity, i.e., no *fundamental* moral status or moral value, over and above the human dignity of the individual real human persons who belong to that social institution. And this point holds necessarily, even if so-called “corporate persons” have had a conventional legal status that has not only consistently belonged to, but also in fact has seriously muddled and tainted, the history of moral, legal, and political thinking about persons, for example in the USA [64].

To be sure, social institutions themselves are constituted by human persons with dignity, and therefore social institutions do indeed take on a certain derivative moral status and moral value. But the dignity of human real persons *inherently overrides* any such social-institutional moral status and moral value. Moreover, and in view of its derivative moral status and value, just because a social institution commands that something is *legally* right or wrong, it does not thereby follow that it is *morally* right (good, virtuous, etc.) or wrong (bad, vicious, etc.). For example, as per Sect. 10, even though digital surveillance of distance workers by business corporations is *legally right*, nevertheless it is obviously morally wrong. As another contemporary example, it is plausibly arguable that the social institution of crime-and-punishment in the contemporary USA, especially including the police and prisons, not only provides a morally scandalous example of rationally unjustifiable “structural racism” and/or “mass incarceration” but also is inherently and systematically authoritarian, coercive, and in violation of human dignity, for anyone who is deemed to be in violation of the laws of the system: hence, while obviously as a system it is legally right, nevertheless equally obviously as a system it is morally wrong [65, 66]. Conversely, something can be *morally right*—say, treating all people with sufficient respect for their dignity—but also *legally wrong*: for example, and again using an example from the USA, under the system of slavery in the USA prior to The Emancipation Proclamation issued by Lincoln in 1863, treating Black people who were slaves *as* equal human persons was in fact *illegal*.

Corresponding to the crucial distinction between morality and legality, is the equally crucial distinction between *deep moral or non-moral responsibility* and *legal reponsibility, aka legal accountability*. As we have seen (i) deep moral or non-moral moral responsibility belongs fundamentally to higher-level or Kantian human real persons, aka moral agents (as opposed to groups of human real persons or to social institutions), by virtue of their choices and/or actions, and in that sense is essentially *first-personal*, (ii) a higher-level or Kantian human real person is deeply morally or non-morally responsible for any choice or intention and/or action that flows from that individual higher-level or Kantian human real person, i.e.,that moral agent, themselves, and (iii) by virtue of the fact that a given choice or intention and/or action flows from that higher-level or Kantian human real person themselves, then the moral value of that choice and/or action, especially including the moral value of some or all of the consequences of that choice and/or action, also directly attaches to that moral agent themselves. By sharp contrast, legal responsibility or accountability concerns only what people can be deemed and held liable for (accused of, blamed for, punished for, etc.) or what social institutions can be deemed and held liable for, by other people or by other social institutions who/that enforce their judgments, policies, rules, and laws by means of coercion, hence it is essentially *second-personal* or *third-personal*. Obviously, then, a higher-level or Kantian human real person can be morally responsible for something without also being legally responsible/accountable for it, and also be legally responsible/accountable for something without also being morally responsible for it. So again, the distinction between the fundamental moral status and value of human real persons (especially higher-level or Kantian human real persons) and the at-best derivative moral status and value of social institutions is crucial to ethics and morality, not to mention social life and politics.

How does all this apply to digital ethics and AI ethics? Our proposal is simply that legal judgments and legal accountability, as applied to the design and/or use of digital technology and computers, ought to conform as closely as humanly possible to the basic moral principles of digital ethics/AI ethics as we have formulated them. In short, we are asserting that *legality* with respect to the design and/or use of digital technology and computers be essentially grounded on broadly Kantian dignitarian *morality* with respect to the design and/or use of digital technology and computers, no matter what the consequences might be for business corporations, judicial systems, the police, governments, etc.

## Conclusion

It is a brute, commonplace fact that nowadays we all live in a thoroughly nonideal natural and social-institutional world that is causally and structurally pervaded by digital technology and computers. But what seems to be far less clearly or widely recognized is that digital technology and computers are our *tools*, not our *masters*, precisely because any kind of domination or mastery over human real persons, especially including coercive authoritarian domination or mastery, is a direct violation of sufficient respect for our human dignity. Therefore, not only philosophers, but also humanity itself, rationally cannot avoid facing up to the task of explicitly formulating, justifying—and, ultimately, generally heeding and following—the basic concepts and principles of dignitarian digital ethics/AI ethics. Indeed, this is a global *existential* project in all the relevant senses of “existential.” So we most earnestly and wholeheartedly hope that our essay will make a direct contribution to that project.

## Footnotes

1 This is a specifically Kantian version of what Frankfurt calls “the doctrine of sufficiency”; see his [25].

2 We will discuss the important distinction between explicit and implicit rational consent in some detail in Sect. 11 below.

3 As opposed to their merely *self-perceived and false human needs*, that is. It might be that someone perceives within themselves an intense need to own a certain luxury automobile, even though they already owns a car that is perfectly adequate to theit true human needs. Therefore, it is not unkind of us not to cater to this self-perceived and false human need. For more on the crucial distinction between true human needs and false human needs, see [9], ch. 3]; and see also Sect. 4 below for a working list of true human needs.

4 See [37,38, 39, and [40]. Savage-Rumbaugh’s research in particular—see, e.g., [37]—is highly controversial. For an alternative view, see [44], esp. pp. 375–379. Our view, which [co-author1] spell outs and defend in [17], chs. 3–4, says that Great apes and perhaps also dolphins are *non-autonomous, non-human real persons* who are morally equivalent to normal human toddlers and other young children. This in turn suggests an argument strategy for those who seek to extend real-person-based legal rights to Great apes and dolphins: Since normal human toddlers and other young children *clearly* have real personhood and dignity, and since Great apes and (perhaps also) dolphins possess the same psychological capacities that ground real personhood and dignity, then it follows that Great apes and (perhaps also) dolphins *also* have real personhood and dignity, and therefore *should also* be accorded the same person-based legal rights. See also [45].

5 See, e.g., books II and III.

6 For the relevant contrast with false human needs, see note 3 above.
